# Editorial: Special Issue “Microbial Diversity of Fermented Food”

**DOI:** 10.3390/microorganisms11051219

**Published:** 2023-05-06

**Authors:** Maria Antoniadou, Theodoros Varzakas

**Affiliations:** 1 Dental School, National and Kapodistrian University of Athens, Thivon 2, 11527 Athens, Greece; 2 Department Food Science and Technology, University of the Peloponnese, 24100 Kalamata, Greece

The goal of this Special Issue was to highlight the diversity of microorganisms associated with fermented foods and their potential key roles in fermentation. Moreover, the aim was to provide information not only on nutritional aspects but also on oral and general health disease prevention and reduction.

In the first paper, by Schiavon et al. [1], next-generation sequencing was employed to determine bacterial diversity in raw cow milk butter processed by traditional fermentation in the Autonomous Province of Trento (Italy). Psychrotrophic genera, mainly *Acinetobacter* and *Pseudomonas*, dominated the traditional mountain butter bacterial community.

Ha et al. [2] reported on traditionally made Kochujang (TMK), a long-term fermented soybean and rice mixture with red pepper and salts, from different provinces of Korea. They found that *Bacillus* spp. (*B. subtilis* and *B. velezensis*) isolated from TMK showed anti-cerebrovascular disease activity and probiotic properties.

Pihurov et al. [3] reviewed wild artisanal cultures (milk and water kefir grains, as well as kombucha—SCOBY), used for to produce fermented beverages, as valuable promoters for metabiotics (prebiotics, probiotics, postbiotics and paraprobiotics) production. They summarized their symbiotic functionality and the ability to obtain valuable bioactive compounds with in vitro and in vivo beneficial functional properties. They discussed new perspectives to develop functional products from the microbial synergy between bacteria—yeast and/or bacteria.

The fourth review paper, by Bezirtzoglou et al. [4], discussed the interaction of gut microbiome and diabetes, focusing on mechanisms connecting gut microbiota with the occurrence of the disorder with special interest of the early clinical signs and symptoms in the oral cavity. The authors addressed the challenge of functional food diet on the maintenance of intestinal health and microbial flora diversity and functionality, thus inhibiting and managing the disease while using oral pre-signs as a motive to incorporate healthier habits for disease control.

Overall dietary research and recommendations on old and new approaches together with a balanced level of action can have potential benefits for public health, diminishing the risk of chronic diseases. Future research efforts should be made on the field of evidence-based dietary solutions of innovative natural formative or technically developed food for both oral and general health.
